# The managed hypertensive: the costs of blood pressure control in a Nigerian town

**Published:** 2012-08-06

**Authors:** Olayinka Stephen Ilesanmi, Olusimbo Kehinde Ige, Akindele Olupelumi Adebiyi

**Affiliations:** 1 Department of Community Medicine, University College Hospital, Ibadan, Nigeria; 2 Department of Preventive Medicine and Primary Care, Faculty of Public Health College of Medicine, University of Ibadan, Ibadan, Nigeria

**Keywords:** Hypertensive, management, financial, cost, time, burden, Nigeria, therapy, patients, drugs, resources

## Abstract

**Background:**

The health systems designed to cater for patients with chronic illnesses like hypertension have not fully evaluated the burden of long term therapy and its effect on patient outcome. This study assessed the financial implication and cost effectiveness of hypertension treatment in a rural Nigerian town.

**Methods:**

A chart review of 250 rural patients with primary hypertension at a regional hospital in Southwest Nigeria was conducted.

**Results:**

The mean age of patients was 61±11.2 years, 59.2% were females, 67% had an income < ₦20,000 ($133.3) monthly. Diuretics and alpha-Methyl Dopa were the most prescribed drugs. The median number of prescribed drugs was two (range1-4). Mean cost of treatment was ₦1440±560 ($9.6±3.7) with 52.8% spending 𢙔 10% of their income on treatment. The most cost effective therapies were Methyl Dopa and Diuretics with Cost-effectiveness ratios of 8 and 12.8 respectively. Patients with co-morbidities, stage 2 hypertension and those on three or four drug regimen had significantly higher treatment costs.

**Conclusion:**

The financial burden of long term antihypertensive therapy appears substantial, cost reduction strategies are needed to optimize hypertension treatment in societies with limited resources. Hypertensive management therefore requires a response adapted to the local context.

## Background

Hypertension is the number one risk factor for cardiovascular disease in sub-Saharan Africa and thus has emerged as a major public health concern [1]. In addition to the large health burden, there is an enormous financial burden associated with the disease [2]. This is because the treatment of hypertension requires an investment over many years to ensure disease-free years among those affected. The economic consequences of this long term therapy often limit the ideals of pharmacologic blood pressure control in many developing countries including Nigeria [2].

In addition to the direct costs in form of treatment related costs there are also indirect costs related to lost man hours and savings among workers due to catastrophic healthcare expenditures. In Nigeria these costs are borne almost entirely by the individuals as the health care financing is still mostly from out of pocket payments [3]. In limited resource settings where chronic disease control is yet to be prioritised and access to health care is limited, cost effective and affordable approaches are needed to meet this challenge [4]. Unfortunately, the number of studies that report these costs is quite small, as few economic analyses relating to management of chronic diseases have been conducted in Nigeria. In addition the health systems designed to cater for patients with chronic illnesses like hypertension have not fully evaluated the burden of long term therapy and its effect on patient outcome. This study therefore assessed the cost of anti-hypertensives, the cost effectiveness of treatment regimens and patient outcomes among rural patients in Southwest Nigeria. Findings would be useful in designing strategies to limit the long-term financial burden on both hypertensive patients and the health system.

## Methods

This was a cross sectional survey of 250 rural patients with primary hypertension attending the General hospital at Igbo-Ora a semi-urban community in Oyo State, South West Nigeria. All patients were from Idere a small town located about 3.5 kilometres from the hospital. Idere is a small town in Ibarapa central Local Government Area of Oyo state. Its local government headquarter is Igboora, which lies about 80 km away from Ibadan, the state capital. It is situated in the rain forest belt of the country. Majority of the residents are the native Yorubas. The main occupations are farming and trading. Social infrastructure available include electricity, mobile telecommunications, several manually operated boreholes, tarred road network, primary health care centres, primary and secondary school [5]. The study involved the chart review of all patients from Idere who had been seen at the hospital over a ten year period (2001-2010). All patients made out of pocket payments for health services and drugs. All prescribed drugs were generic.

### Measures

Stage of hypertension at diagnosis was according to the JNC 7 classification [6]. Patients with systolic blood pressure of 140–159 mmHg or diastolic 90–99 mmHg were classified as stage 1 while patients with systolic blood pressure of ≥ 160 mmHg or diastolic ≥ 100mm Hg were classified as having stage 2 hypertension The primary outcomes used was the achievement rates of target blood pressure (i.e BP < 140/90 mmHg or < 130/80 mm Hg for diabetics). Persistence with therapy according to the International Society for Pharmacoeconomics & Outcomes research was defined as the continuing use in time of the prescribed therapy [7]. This was recorded as documented from patient's history in the case records.

### Cost Analysis

The direct cost examined was limited to the cost of antihypertensive medication. Cost was calculated as a function of the dosage prescribed and the prices in the hospital or community pharmacies as of September, 2010. The monthly cost of a 30-day antihypertensive supply based on the recommended daily dose was computed. Indirect costs were the cost of transportation to the hospital. The total cost was the sum of the both the direct and indirect costs.

Mono-therapy was defined as use of a single medication containing only one antihypertensive agent. Combination drug therapy was defined as use of multiple antihypertensive agents.

Cost-effectiveness ratios [8] were estimated as the average treatment cost per patient reaching the target blood pressure. The cost-effectiveness relationship was calculated as the ratio of the monthly mean cost to the proportion of patients with controlled HBP, for each pharmacological group of drugs prescribed. The CER was adjusted for hypertension staging at diagnosis.

A relative CER was also described for each drug group, using the CER of the most cost-effective therapy as denominator (i.e. relative CER = CER of therapy/CER of most cost-effective therapy in the study) [2] CER = mean monthly cost /% patients with controlled Blood Pressure, RCER = CER/CER of most cost effective drug.

**Data Analysis:** mean costs were compared with the student t test at a level of significance of 5%.

## Results

The mean age of patients was 61±11.2 years. More than half 167(66.8%) were 60 years and above and 148 (59.2%) were females. Many of the patients were traders 142(56.8%). The mean household income was ₦14,300 ± ₦2, 940($95.3±$19.6). (Table 1).

**Table 1:** Socio-demographic characteristics of patients

Variables	Frequency (%)
Sex	
Male	102 (40.8)
Female	148 (59.2)
Age (years)	
< 40	8 (3.3)
40-49	32 (12.8)
50-59	43 (17.2)
≥60	167( 66.8)
Occupation	
Farmer	63( 25.2)
Trader	142 (56.8)
Artisan	17( 6.8)
Civil servant	28 (11.2)
Household monthly income^*^	
Less than ₦20,000 ($133.3)	167( 67.0)
₦20,000 ($133.3)and above	83 (33.0)

Household income was estimated in a study by O K Ige and C. Nwachukwu [23]. The conversion rate was ₦150 =$ 1 when the study was conducted

### Clinical profile of patients

The majority of patients had Stage 2 hypertension had diagnosis and 39(15.6%) had other chronic illnesses other than hypertension (Table 2)

**Table 2:** Clinical profile of patients

Variables	N = 250(%)
Hypertension staging at diagnosis	
Stage 1	44(17.6)
Stage 2	206(82.4)
Co-morbidities+	
Present	39(15.6)
Absent	211(84.4)

+ Co-morbidities identified were Diabetes and Obesity

### Prescribed antihypertensive regimen

**Figure 1:**
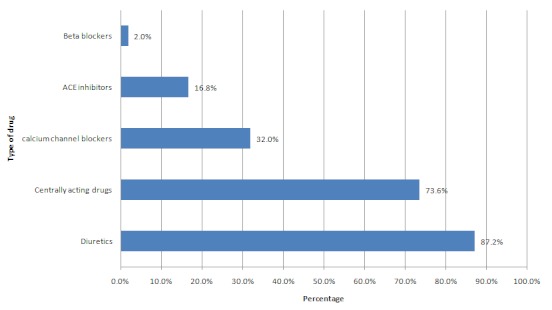
Prescribed anti-hypertensive

The number of drugs prescribed ranged from 1-4 (median 2). Only 32(12.8%) had monotherapy, 157(62.8%) had two drugs, 58(23.2%) three drugs and 3(1.2%) four drugs prescribed. The median duration of treatment was 12 months (range 1- 91months). The classes of anti-hypertensives commonly prescribed are shown in Figure 1. Diuretics and Alpha methyl dopa (centrally acting) were the most often prescribed anti-hypertensive.

### Direct monthly cost of treatment and cost effectiveness

Table 3 shows the monthly costs and the cost effectiveness of the frequently prescribed drugs. The median monthly cost of drugs was ₦900 (range ₦300 - ₦3,000) $6(range$2-$20). The most cost-effective drug was Methyl Dopa with an average monthly cost of ₦800 ($5.3), and CER of 8. Two drug therapies were more cost effective than three drug regimens. Table 4 shows the transportation costs. The round fare to the hospital was ₦120. Close to half were given monthly appointment 114 (45.6%).

**Table 3:** Adjusted Cost effectiveness of common Anti hypertensive therapy

Drugs	Frequency (%)	Monthly Cost	Controlled BP Freq (%)	Average CER	RCER
CA alone	10(4.0)	₦800.00($5.3)	10(100)	8	1.0
Diuretics alone	17(6.8)	₦300.00($ 2)	4(23.5)	12.8	1.6
D+ CCB	29(11.6)	₦1200.00($ 8)	11(37.9)	31.7	4.0
D+ CA	105(42)	₦1200.00($ 8)	39(37.1)	32.3	4.0
D + ACEI	8(3.2)	₦1300.00($8.6)	3(37.5)	34.7	4.3
D + BB	5(2.0)	₦1400.00($9.3)	2(40)	35	4.4
D + CCB + CA	31(12.4)	₦2000.00($13.3)	8(25.8)	77.5	9.7
D + CA + ACEI	23(9.2)	₦2100.00 ($14)	3(13.0)	161.5	20
CA + CCB	8(3.2)	₦1700.00($11.3)	0(0)	-	-
Others^1^	14(5.6)	₦3000.00($20)	4(28.)	750	93.75

D= Hydrochlorothiazide/Amiloride (Diuretics), CCB= Nifedipine (Calcium channel blockers), ACEI= Lisinopril (Angiotensin converting enzyme inhibitor), BB= Atenolol (β-blockers), CA= Alpha methyldopa (Centrally acting drugs); ^1^Others are ACEI alone, CCB only, D + ACEI + CA + CCB, D + ACEI + CCB, CCB + CA + ACEI

1 Others are ACEI alone, CCB only, D + ACEI + CA + CCB, D + ACEI + CCB, CCB + CA + ACEI

**Table 4:** In-direct costs (transportation cost)

Frequency of appointment	N (%)	Transportation cost/month
Weekly	36 (14)	₦480.00 ($3.2)
Fortnightly	77 (30.8)	₦240.00($1.6)
Monthly	114 (45.6)	₦120.00($0.8)
Two monthly	23 (9.2)	₦60.00($0.4)

### Total cost implication of treatment

The mean total cost of treatment was ₦1440±560 ($9.6±3.7), the mean proportion of the household income spent on treatment was 11.1% ± 4.2%, 132(52.8%) spent greater than 10% of their total household income on treatment.

### Treatment outcome

Less than half (47.6%) were persistent with prescribed drugs and appointments (55.2%- mono-therapy and 45.9%- combination therapy (p = 0.34)). Successful BP control was recorded in 33.6% overall (48.3% -mono-therapy and 32.1%-combination therapy; p = 0.084).

### Comparison of mean treatment costs

The mean total cost of treatment was significantly higher for those who had co-morbities, higher BP at diagnosis and those on 3 or 4 drug regimens. Higher mean cost was also significantly associated with a higher percentage of the total household income being spent (P < 0.05). The mean cost of treatment did not vary significantly by age, sex or income of the patients. The mean cost was not significantly associated with adherence with prescribed treatment (Table 5).

**Table 5:** Comparison of mean treatment costs

Variable	N(%)	Mean cost ±S.D (₦)	T test	P value
**Sex**				
Male	102(40.8)	1590.20 ± 491.22($ 10.6±3.2)	0.08	0.936
Female	148(59.2)	1595.68 ± 555.72($10.64±3.7)		
**Age groups**				
<60yrs	83(33.2)	1595.90 ± 403.21($10.63±2.7)	0.058	0.954
60 and above	167(66.8)	1592.22 ± 583.07($10.63±3.8)		
**Co-morbidities**				
Yes	39(15.6)	1815.90 ± 613.09($12.1±4.1)	2.899	0.004
No	211(84.4)	1552.32 ± 503.32($10.3±3.4)		
**Staging at diagnosis**				
Grade 1	44(17.6)	1267.27 ± 475.07($8.4±3.2)	4.689	<0.001
Grade 2	206(82.4)	1663.11 ± 515.06($11.1±3.4)		
**Number of drugs**				
Two or less	189(75.6)	1352.06 ± 321.95($15.6±1.9)	21.313	<0.001
Three or more	61(24.4)	2341.31 ± 293.03($10.6±3.7)		
**Persistence to therapy**				
Yes	119(47.6)	1589.75 ±561.80($10.6±3.7)	0.105	0.917
No	131(52.4)	1596.80 ±500.17($10.6±3.3)		
**Income groups**				
<20,000	222(88.8)	1573.33 ±529.67($10.5±3.5)	1.698	0.091
≥20,000	28(11.2)	1752.86 ±507.96($11.7±3.4)		
**Percentage of household income spent on treatment**				
<10%	118(47.2)	1242.37 ± 322.81($8.3±2.2)	12.719	<0.001
≥10%	132(52.8)	1907.27 ± 478.80($12.7±3.2)		
**BP control**				
Yes	84(33.6)	1471.67 ± 447.47($9.8±2.9)	2.618	0.009
No	166(66.4)	1655.06 ± 557.45($11.0±3.7)		

## Discussion

This study evaluated treatment cost for rural patients with hypertension and the cost effectiveness of prescribed antihypertensive treatment. Many patients were prescribed two or more drugs with diuretics and centrally acting drugs being the most commonly prescribed drugs. Although alpha-Methyl Dopa is not included in recent treatment guidelines for managing hypertension at the community level in Sub Saharan Africa [9] it continues to be prominent in the treatment regimen of hypertension as documented in other African surveys [10–12]. This is probably due to the low cost and observed effectiveness. Even though the number of patients on a-Methyl Dope as mono therapy was small in comparison to other drug types, it emerged as the most cost effective drug in this cohort of patients. The standardized monthly cost of antihypertensive drug therapy was lower for diuretics in comparison with any other drug. Similar low costs relating to diuretics have been described in other studies [13–15]. Diuretics as fixed dose combinations (FDC) of Hydrochlorothiazide and Amiloride also emerged as one of the most cost effective treatment option, replicating other findings [16]. Guidelines for Sub-Saharan Africa have also re-affirmed the efficacy of diuretics and diuretic-based combinations over other classes of antihypertensive drugs [17]. The CER for FDC diuretic of 12.8 was much lower than the 42.9 reported in another Nigerian study conducted in a tertiary hospital in an urban setting [10]. The observed differences CER might be attributable to the different study settings.

Persistence with drug therapy among this rural population was 47.6% much lower than the 82.5% previously documented among urban patients in Nigeria [2]. Even though those on mono-therapy were more often persistent with therapy this was not significantly so. This varies from the findings of other African studies in which cost appeared as a major factor in compliance with treatment among hypertensive patients [18–19]. The rates of blood pressure control was also low, only about a third of patients achieved the target BP, higher control rates were observed for patients on mono-therapy as shown by previous studies [2, 20]. Some population studies have also reported hypertension control rates as low as 53.1% for the US, 41.0% for Canada, 33.6% for Germany and 29.2% for the UK [21].

The economic burden of hypertension treatment was significant, about half of the patient were spending a tenth or more of their income on health care related expenses. The mean cost of treatment was significantly higher for those who had co-morbidities, higher BP at diagnosis and those on 3 or 4 drug regimens. Similar relations showing a higher total cost for patients with co-morbid conditions and patients on combination therapy have been observed in another work [22]. It seems patients with higher risk also bear the heaviest financial burden. The utilization of cheaper yet equally effective diuretics based drug combinations may thus be most beneficial.

## Conclusion

Thiazide diuretics are still the backbone of hypertension treatment especially in low resource settings even as mono-therapy. Hypertensive management therefore requires a response adapted to the local context. Proven solutions are needed to optimize the application of treatment guidelines in less developed societies with limited resources and to explore cost reduction strategies in the management of hypertensive in a poor resource setting. Alpha Methyl Dopa is a useful drug in the management of Hypertension though it was not included in the guideline. The cost of health care is probably underestimated as other health care related costs such as those for laboratory tests and hospital admissions could not be ascertained. In assessing cost effectiveness of antihypertensive the influence of patients’ compliance and persistence with treatment were not considered. Another limitation is the small number of patients on other combinations of antihypertensive apart from those listed which precluded them from analysis, larger numbers might have allowed for more valid comparison. Inspite of these limitations the study provides useful data on individual expenditure on hypertension treatment in Nigeria.
